# Mandibular condyle tissue reaction to low intensity pulsed ultrasound in young adult rats: Micro computed tomographic and histomorphometric datasets

**DOI:** 10.1016/j.dib.2022.108185

**Published:** 2022-04-17

**Authors:** Yasamin Hadaegh, Tarek H. El-Bialy

**Affiliations:** University of Alberta1,^2^, Canada, 7-020D Katz Group Centre for Pharmacy and Health Research, University of Alberta, Edmonton, Alberta, T6G 2E1, Canada

**Keywords:** Growth modification of the mandible, Temporomandibular joint, LIPUS (low intensity pulsed ultrasound), Microstructural evaluation, Endochondral bone growth, Chondrogenesis, Osteogenesis

## Abstract

Mandibular condyle (MC) in postnatal life, grows mainly by endochondral bone growth which is a multistep process and the condylar cartilage plays a vital role in its regional adaptive growth. Hence, for determining the exact effect of a treatment such as low Intensity pulsed ultrasound (LIPUS) on the MC growth in animal models, it is important to reliably and reproducibly detect changes at different tissue levels and correct regions of the condyle. To this aim, micro computed tomography (µCT), as well as Alcian Blue-Pas staining, in vivo flourochrome labeling via calcein green, and Goldner's Trichrome staining on proper decalcified and undecalcified sections was performed for the harvested samples from young adult rats. Standardized procedures were used to determine volumes or regions of interest for microstructural evaluations in the middle and posterior areas of the MC. In the condylar cartilage, the thickness of fibrous, proliferative, chondroblastic, and total fibrocartilage layers; also the cell population in proliferative and chondroblastic layers were precisely measured. On the other side, using accurate methods percentage of calcifying cartilage and newly formed bone areas/bone area, bone volume fraction and specific surface, trabecular number, thickness, and separation, degree of anisotropy, bone mineral density; furthermore, the amount of actual endochondral bone growth and the osteoid thickness were quantified in subchondral cancellous bone subjacent to condylar cartilage. Data provided herein present the robust µCT and histomorphometric evaluations of the control and LIPUS treated adult MCs at cartilage and bone level. Data also highlights the difference in tissue response to the stimuli between the middle and posterior regions of the condyle. Further interpretation of these datasets can be found in https://doi.org/10.1016/j.bonr.2021.101122[1].

## Specifications Table

**Table:** 

Subject	Dentistry, Oral Surgery and Medicine
Specific subject area	Microstructural evaluation of the mandibular condyle following growth stimulation
Type of data	Figure Table
How the data were acquired	Right and left hemi-mandibles were scanned via a high-resolution compact fan-beam tomogram (µCT, SkyScan 1072, Aartselaar, Antwerp, BE) and associated software (Version 2.6.0). Scanned images were reconstructed using NRecon© (Version 1.4.4) from SkyScan®. Reconstructed images were analyzed using CT Analyser (Version 1.6.1.0, Skyscan N.V. Kontich, BE).Two volume of interest in the middle and posterior regions of the MC were determined. Densitometric and morhometric changes of the trabecular bone subjacent to condylar cartilage were evaluated. Furthermore, serial decalcified and undecalcified sections were respectively obtained from right and left condyles at midsagittal plane. Photomicrographs of Alcian Blue-Pas and Goldner's Trichrome stained slides were taken using a Leica fluorescent digital microscope with a CCD Digital camera (Leica, Wetzlar, Germany). The calcein green labels produced by in vivo flourochrome injection at 7 and 28 days of the experiment, were visualized under epifluorescence illumination and photomicrographs were taken using an Olympus Fluo View 1000 Inverted IX81 microscope. Image processing analyses were done using RS Image software (Version 1.73, Photometrics, Roper Scientific, Tucson, AZ, USA) and histomorphometric evaluations were performed in the middle and posterior regions of the condyle.
Data format	Analysed Raw
Description of data collection	Bone volume fraction and specific surface, trabecular number,thickness, and separation, degree of anisotropy, and bone mineral density were quantified to gather the micro computed tomograhic data. Histomorphometric data collection was performed as follows:The thickness of fibrous, proliferative, chondroblastic, and total fibrocartilage layers; the cell population in proliferative and chondroblastic layers; bone volume fraction and percentage of calcifying cartilage remnants and newly formed bone areas/bone area were measured on Alcian Blue/Pas stained slides. Actual endochondral bone growth were determined by measuring the distance between the two calcein lables and the osteoid thickness were quantified on Goldner's Trichrome stained slides.
Data source location	• University of Alberta • Edmonton, Alberta • Canada
Data accessibility	Mendeley Data, V1, doi: 10.17632/bz8tcv6dgh.1 https://data.mendeley.com/datasets/bz8tcv6dgh/1
Related research article	Y. Hadaegh; H. Uludag; D. N. Dederich; T. H. El-Bialy, The effect of low intensity pulsed ultrasound on mandibular condylar growth in young adult rats. J. Bone Rep. 15 (2021) 101122. https://doi.org/10.1016/j.bonr.2021.101122

## Value of the Data

• To determine the exact effect of treatments on the MC growth, it is important to reliably and reproducibly detect changes at different tissue levels and correct regions of the condyle. As presented in the data, using µCT and specialized staining techniques on proper decalcified and undecalcified sections; also precise methods to quantify the microstructural changes of the condylar cartilage and subchondral cancellous bone, in the middle and posterior regions, such an evaluation became possible.
• These data sets are of particular interest to researchers who are working on growth modification of the MC. In addition, they can be beneficial for the wider scientific community of bone and cartilage.
• The methods described here are specifically apt to be used for evaluating microstructural changes of the MC, especially in rodents, due to application of therapeutic or growth promoting drugs and techniques or while growth and development. Nevertheless, they can be used for similar evaluations of endochondral bone growth, trabecular bone remodeling, and cartilage or bone formation in general.
• The data provided in the tables can be used for comparison in similar experiments which evaluate the effect of an altering factor on the results. Thus, the time and resources can be saved and the animal loss would be less.

## 1 Data Description

As a supplementary data related to the research article https://doi.org/10.1016/j.bonr.2021.101122 [1], herein detailed microcomputed tomographic and histomorphometric evaluations of the control and LIPUS treated adult rat MCs at cartilage and bone level as well as in the middle and posterior regions of the condyle are provided.

**Fig. 1:**
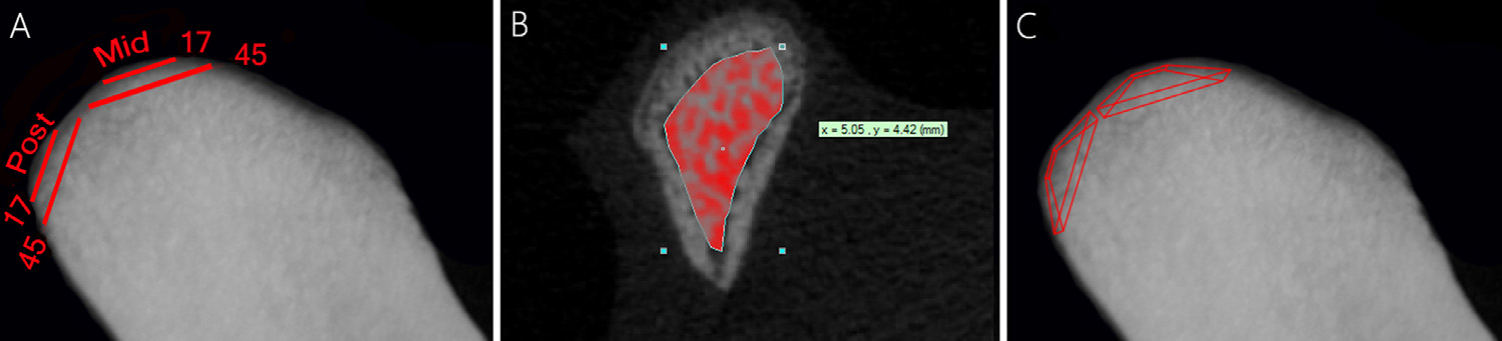
Illustration of the method to locate the volume of interest for µCT analysis of the trabecular bone subjacent to the condylar cartilage: A) The uppermost point of the middle and posterior regions of the condyle were determined, then from the reference line, 80 slices with an increment of 18 µm were scanned and the 17th to 45th slices were selected for each region. B) In each slice, the whole trabecular bone was selected manually. C) Final volume of interest evaluated for morphometric and bone mineral density analysis.

**Fig. 2:**
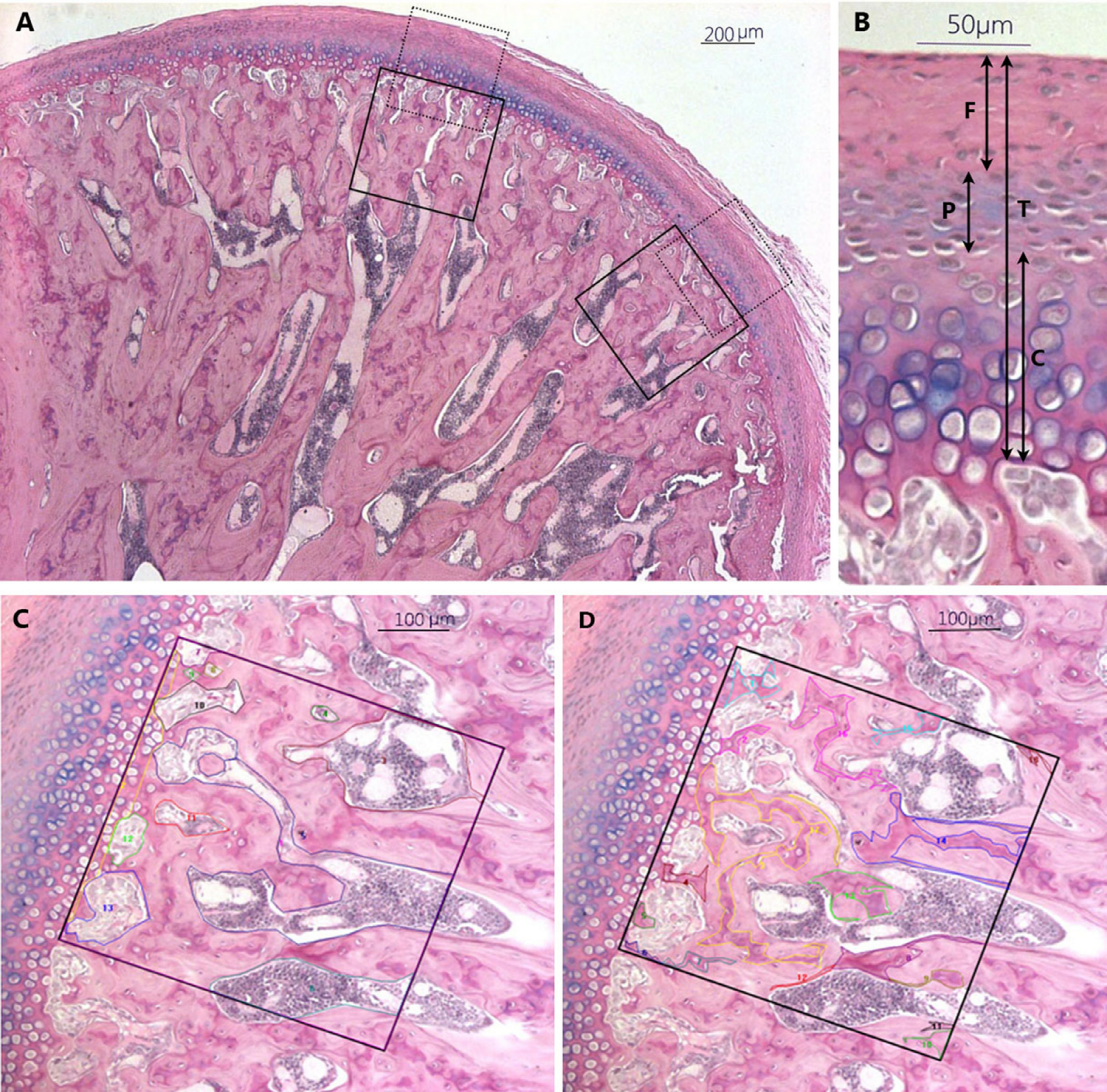
Demonstration of histomorphometric evaluation on Alcian Blue/PAS stained MCs (cartilage: blue, calcifying cartilage matrix and new bone: distinct magenta, mature bone: pale pink): A) The surface of condylar cartilage was equally divided into three parts: anterior, middle and posterior. Two measurement frames were located at the middle third of the middle and posterior thirds. One for the measurement of layers and cell counting (doted, 450 × 450µm) at x560 magnification and x20 objective magnification (B), and the other for calculating bone volume fraction and percentage of newly formed bone and calcifying cartilage area / bone area (solid, 500 × 500µm) at x280 magnification and x10 objective magnification (C & D). B) For measuring the thickness of fibrocartilage layers, i.e., fibrous layer (F), proliferative layer (P), chondroblastic layer (C), total fibrocartilage layer (T), six equally distributed lines parallel to each other and perpendicular to the outer contour of the articular surface were quantified and averaged for each region of each section. C) To calculate BV/TV% [100 x (bone area of subchondral trabecular bone/tissue area)], tissue area (0.5 × 0.5 mm square – hypertrophic area invaded in erosive zone) and bone area (tissue area- bone marrow area) were measured. D) To determine the percentage of [100 x] calcifying cartilage and newly formed bone areas/bone area, distinctive magenta areas were also measured.

**Fig. 3:**
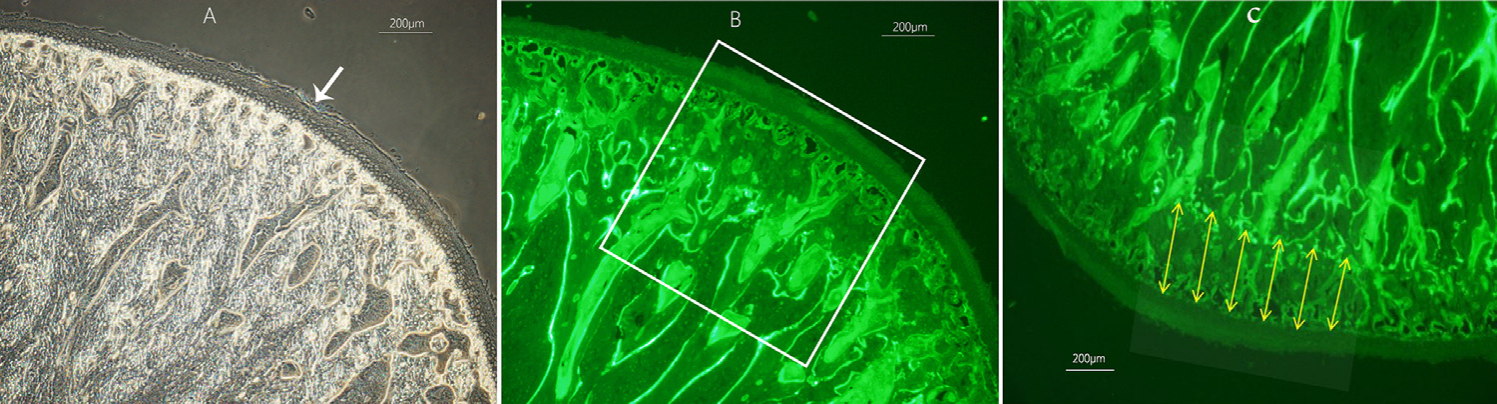
Illustration of histomorphometric evaluation on undecalcified sections of the MC to estimate the amount of endochondral bone growth via in vivo flourochrome labeling: A) The arrow points to the midpoint (top) of the condylar surface demarcated with tissue marker dye, prior to sectioning at midsagittal plane. B) Under epifluorescence illumination, a square of 940 µm x 940 µm at x82 magnification in the middle region of each section was used for evaluation. C) To measure the distance between calcein labels, six equally distributed lines parallel to each other and perpendicular to the outer contour of the articular surface between the upper most points of the first and second labels were quantified and averaged.

**Fig. 4:**
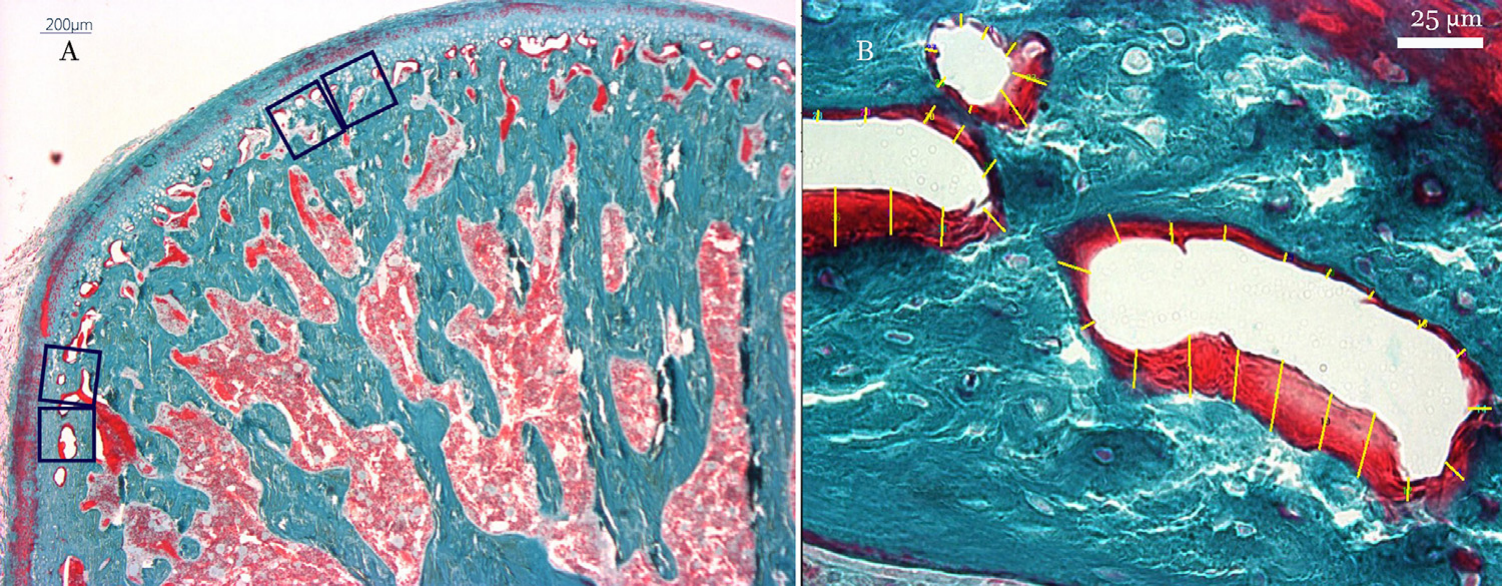
Demonstration of the method for evaluating the changes in osteoid thickness, on undecalcified sections of the MC stained by Goldner's Trichrome (mineralized bone tissue: greenish blue, Osteoid: red): A) Two successive measurement frames of 240µm*240µm squares were located at the interface of cartilage and subchondral cancellous bone in the middle and posterior regions of the condyle. B) For every measurement field at x 940 magnification, 10 to 60 measurements were obtained at fixed intervals along the osteoid surface and osteoid thickness was calculated by averaging the osteoid thickness values.

How these evaluations have been performed are demonstrated in figures (1-4).

Dahlberg's formula [2] was used to calculate the error of measurement. Intra rater reliability for 6 randomly selected animals for each measured variable was tested using an intra-class correlation coefficient (ICC) test. This data and the results for each measured variable is shared in https://data.mendeley.com/datasets/bz8tcv6dgh/1 excel files (1-4) sheet one.

All the evaluations were done twice with an interval of two weeks. There was no significant difference between the two registrations (using paired t-test) (for all p>0.05); thus, the mean value representing each hemi-mandible for all the evaluated parameters was used for statistical analysis described below. This data is shared in https://data.mendeley.com/datasets/bz8tcv6dgh/1 excel files (1-4) sheet two.

For all the measured variables, to compare the groups when compensating for correlation between the outcomes, considering either laterality (left and right mandibular condyles) and/or location (middle and posterior regions), generalized estimating equation (GEE) was used to analyze the data. To perform two-by-two comparison when considering the multiple comparisons, Bonferroni method was employed. However, for comparison of the groups on the amount of endochondral bone growth, considering two independent samples and the fact that equality of variance and normality were met, independent sample t-test was used. To present data, mean, standard deviation (SD), median and inter quartile range (IQR) were used. A P-value less than 0.05 was considered statistically significant. All statistical analysis was performed by SPSS (version 21.0, IBM Co., Chicago, IL, USA) and presented in tables (Table 1, Table 2, Table 3, Table 4).

**Table 1:** The comparison of densitometric and morphometric bone parameters of subchondral cancellous bone in experimental and control groups evaluated by µCT analysis.

		LIPUS		Control			
Response	Location	Mean ± SD	Median (IQR)	Mean ± SD	Median (IQR)	Mean difference	P-value
BV/TV (%)	Middle	65.067 ± 1.479	64.867 (64.206 to 66.307)	66.452 ± 1.744	66.759 (65.38 to 67.554)	–1.453	0.046†
	Posterior	61.805 ± 3.91	62.772 (61.544 to 63.839)	60.283 ± 4.644	60.722 (59.066 to 63.956)	1.522	0.387†
							<0.001§
	Total	63.436 ± 3.353	64.173 (62.621 to 65.23)	63.367 ± 4.661	64.801 (60.722 to 66.759)	0.069	0.579⁎
BS/BV (mm-1)	Middle	51.911 ± 2.552	52.461 (51.176 to 53.426)	48.013 ± 4.927	49.496 (44.744 to 51.499)	3.895	0.031
	Posterior	52.901 ± 5.369	53.491 (51.579 to 57.057)	49.046 ± 5.29	50.962 (44.514 to 52.099)	3.855	0.108
							0.020
	Total	52.406 ± 4.179	52.711 (51.374 to 54.253)	48.529 ± 5.056	50.411 (44.514 to 51.695)	3.877	0.171
Tb.Th (mm)	Middle	0.072 ± 0.002	0.072 (0.072 to 0.073)	0.076 ± 0.006	0.074 (0.072 to 0.08)	–0.004	0.058
	Posterior	0.075 ± 0.004	0.074 (0.073 to 0.075)	0.078 ± 0.006	0.076 (0.075 to 0.081)	–0.003	0.088
							<0.001
	Total	0.074 ± 0.003	0.073 (0.072 to 0.074)	0.077 ± 0.006	0.075 (0.074 to 0.081)	–0.003	0.188
Tb.Sp (mm)	Middle	0.071 ± 0.011	0.067 (0.066 to 0.071)	0.076 ± 0.012	0.071 (0.067 to 0.085)	–0.005	0.364
	Posterior	0.082 ± 0.02	0.072 (0.07 to 0.082)	0.092 ± 0.018	0.084 (0.077 to 0.109)	–0.01	0.229
							<0.001
	Total	0.077 ± 0.017	0.07 (0.067 to 0.078)	0.084 ± 0.017	0.078 (0.071 to 0.093)	–0.007	0.094
Tb.N (mm-1)	Middle	9.378 ± 0.298	9.213 (9.163 to 9.69)	8.57 ± 0.99	8.985 (7.888 to 9.253)	0.808	0.019
	Posterior	8.363 ± 0.847	8.655 (8.269 to 8.806)	7.745 ± 1.009	7.904 (7.261 to 8.621)	0.618	0.134
							<0.001
	Total	8.871 ± 0.811	9.077 (8.655 to 9.218)	8.157 ± 1.069	8.574 (7.414 to 8.985)	0.714	0.131
DA (ratio)	Middle	1.371 ± 0.212	1.321 (1.252 to 1.412)	1.719 ± 0.505	1.539 (1.348 to 1.921)	–0.348	0.017
	Posterior	2.12 ± 0.533	1.991 (1.74 to 2.393)	2.176 ± 0.741	1.944 (1.683 to 2.622)	–0.056	0.797
							<0.001
	Total	1.745 ± 0.551	1.645 (1.321 to 1.991)	1.947 ± 0.665	1.769 (1.515 to 2.292)	–0.202	0.196
BMD (mg/cm3)	Middle	2.507 ± 0.596	2.158 (2.061 to 3.205)	3.395 ± 0.388	3.39 (3.221 to 3.717)	–0.808	<0.001
	Posterior	2.295 ± 0.757	2.167 (1.629 to 2.851)	2.797 ± 0.499	2.763 (2.463 to 3.09)	–0.502	0.071
							0.636
	Total	2.401 ± 0.681	2.158 (1.887 to 2.996)	3.096 ± 0.534	3.221 (2.71 to 3.469)	–0.695	0.006

† Based on GEE considering the correlation in the responses.

§ Comparison of posterior and middle regions based on GEE.

⁎ Based on GEE considering the correlation in the responses and adjusted for the effect of location and laterality.

**Table 2:** The comparison of histomorphometric parameters evaluated on decalcified sections stained with Alcian blue/PAS in experimental and control groups.

		Control		LIPUS			
Response	Location	Mean ± SD	Median (IQR)	Mean ± SD	Median (IQR)	Mean Difference	P-Value
Total fibrocartilage thickness (µm)	Middle	170.981 ± 28.267	169.839 (158.889 to 178.730)	233.049 ± 63.762	216.373 (182.690 to 265.357)	62.068	.004*
	Posterior	183.279 ± 16.892	186.222 (176.313 to 191.607)	249.464 ± 69.551	236.844 (185.188 to 273.110)	66.185	.002*
							.562§
	Total	177.130 ± 23.375	178.730 (162.806 to 186.235)	241.257 ± 65.483	227.014 (184.302 to 269.233)	64.157	<0.001†
Fibrous layer thickness (µm)	Middle	49.717 ± 9.893	49.804 (46.007 to 55.807)	70.642 ± 26.906	66.520 (50.534 to 78.106)	20.925	.016
	Posterior	62.108 ± 11.680	62.712 (57.372 to 68.658)	94.112 ± 38.676	79.358 (63.680 to 112.823)	32.004	.009
							.097
	Total	55.912 ± 12.258	57.372 (48.671to 63.464)	82.377 ± 34.590	72.551 (53.753 to 98.738)	26.465	<0.001
Proliferative layer thickness (µm)	Middle	41.928 ± 10.738	39.336 (33.245 to 50.189)	59.162 ± 15.986	56.130 (45.393 to 69.133)	17.234	.004
	Posterior	44.088 ± 7.914	45.142 (36.255 to 50.150)	61.692 ± 19.155	54.109 (50.135 to 78.579)	17.604	.005
							.735
	Total	43.008 ± 9.180	42.627 (35.794 to 50.150)	60.427 ± 17.220	54.109 (46.666 to 73.856)	17.419	<0.001
Chondroblastic layer thickness (µm)	Middle	86.613 ± 19.720	79.495 (72.311 to 102.361)	110.836 ± 24.375	108.765 (98.837 to 111.250)	24.223	.013
	Posterior	81.820 ± 18.893	73.800 (65.955 to 100.733)	95.825 ± 17.488	90.438 (83.606 to 116.155)	14.005	.086
							.095
	Total	84.216 ± 18.820	76.985 (67.570 to 100.733)	103.331 ± 22.036	101.019 (85.385 to 113.702)		0.004
Cell population in Proliferative layer	Middle	165.125 ± 17.455	159 (151.5 to 180.5)	201.5 ± 32.694	203 (184 to 213)	36.375	.001
	Posterior	172.5 ± 11.880	175.5 (164 to 180.5)	229.3 ± 63.113	203.5 (196 to 218)	56.8	.003
							.192
	Total	168.812 ± 14.918	170 (154 to 180.5)	215.4 ± 50.956	203 (189 to 215.5)	46.588	<0.001
Cell population in Chondroblastic layer	Middle	138.5 ± 28.319	131.5 (118.5 to 159.5)	185.5 ± 28.632	185 (160 to 194)	47	.000
	Posterior	111.25 ± 32.840	127.5 (84 to 133)	136 ± 45.77	125.5 (116 to 166)	24.75	.157
							.002
	Total	124.875 ± 32.796	127.5 (110.5 to 140.5)	160.75 ± 45.005	162.5 (125.5 to 188)	35.875	0.004
BV/TV (%)	Middle	69.986 ± 4.346	68.657 (67.255 to 71.599)	61.909 ± 8.183	63.197 (56.584 to 68.069)	–8.077	.005
	Posterior	58.880 ± 12.948	55.428 (53.274 to 63.793)	64.130± 10.291	64.007 (57.649 to 72.456)	5.25	.320
							.573
	Total	64.433 ± 10.952	67.255 (55.428 to 70.545)	63.020 ± 9.120	64.007 (57.116 to 68.907)	–1.413	0.670
Remnants of calcifying cartilage &							
newly formed bone area/bone area (%)	Middle	15.473 ± 6.334	14.379 (11.679 to 17.293)	24.702 ± 6.269	24.745 (21.993 to 31.395)	9.229	.001
	Posterior	19.786 ± 6.906	18.140 (14.034 to 25.780)	22.074 ± 4.933	22.164 (18.3767 to 26.1829)	2.228	.401
							.272
	Total	17.630 ± 6.777	16.082 (12.547 to 21.642)	23.388 ± 5.653	22.672 (18.592 to 27.119)	5.758	0.005

*Based on GEE and adjusted for multiple comparison by Bonferroni method.

§ Comparison of the posterior and middle regions in LIPUS group based on GEE.

† Based on GEE considering the correlation in the responses and adjusted for the effect of location.

**Table 3:** The amount of actual endochondral bone growth (µm) in experimental and control groups.

	Control	LIPUS	P†
Mean±SD	264.894±49.899	318.594±58.358	0.002
Median (IQR)	265.139(223.146 to 307.233)	306.2744 (272.071 to 369.756)	

† Based on independent sample t-test.

**Table 4:** The comparison of osteoid thickness (µm) in experimental and control groups.

	LIPUS		Control		
Location	Mean ± SD	Median (IQR)	Mean ± SD	Median (IQR)	P†
Middle	8.38 ± 0.87	8.3 (7.62 to 9.16)	5.65 ± 0.99	5.36 (5.14 to 5.71)	<0.001
Posterior	8.06 ± 1.34	8.28 (6.74 to 9.15)	6.37 ± 0.97	6.59 (5.45 to 7.02)	0.002
					0.560§
Total	8.22 ± 1.11	8.3 (7.37 to 9.16)	6.01 ± 1.01	5.57 (5.34 to 6.65)	<0.001*

† Based on GEE considering the correlation in the response.

§ Comparison of the posterior and middle regions.

* Adjusted for the effect of location.

## 2 Experimental Design, Materials and Methods

Following 10 days’ acclimatization period, eighteen 120-day-old female rats (control (n=8) and LIPUS treated (n=10)) were kept under general anesthesia 20 minutes each day for 28 consecutive days, during which LIPUS applied to the temporomandibular joints (TMJs) of the LIPUS group bilaterally. Then mandibles were dissected and fixed in a formalin solution. Coding has been performed to address blinding in subsequent assessments.

### 2.1 Micro computed tomographic evaluation

Rat hemi-mandibles were mounted in cylindrical specimen holders (polypropylene, outer diameter: 29 mm, wall thickness: 1 mm), secured with synthetic foam and completely submerged in fixation fluid. Scanning was performed via a high-resolution compact fan-beam tomogram (µCT, SkyScan 1072, Aartselaar, Antwerp, BE) and associated software (Version 2.6.0) at a resolution of 18μm using an x-ray source potential of 85kV, amperage of 290μA, and power of 25W through 180^o^ with a rotation step of 0.5^o^, to produce serial cross-sectional images. An aluminum filter of 1.0 mm thickness was used, and three projections for each scanned section were averaged. Scanned images were saved in *.tiff format. Scion Image, beta 4.0.2 (Scion Image Corporation, USA) was used to median-filter the raw image data to reduce noise. The filtered image data was rendered in three dimensions. Using this orientation, the 2-D image stacks were exported to a commercial image analysis package (IP-PLUS, Media Cybernetics, Bethesda, MD, USA). Finally, the images were reconstructed using NRecon© (Version 1.4.4) from SkyScan®. Reconstructed images were analyzed using CT Analyser (Version 1.6.1.0, Skyscan N.V. Kontich, BE).

For evaluating the changes of the trabecular bone subjacent to condylar cartilage, a modified method which has been previously explained by Xiong et al. [3] was used to locate the volume of interest (Fig. 1). Local adaptive threshold algorithms with prethresholding between 48 and 225 were used during the evaluation of all specimens. Standard bone microstructural parameters [3], [4], [5], namely Bone Volume Fraction (Bone Volume / Tissue Volume) (BV/TV (%)), Bone Specific Surface (Bone Surface to volume ratio) (BS/BV(mm-1)), Trabecular Number (Tb.N (mm-1)), Trabecular Thickness (Tb.Th (mm)), Trabecular Separation (Tb.Sp (mm)), and Degree of Anisotropy (DA (ratio)), were evaluated using model independent, three dimensional morphometric analysis. In addition, to evaluate bone mineralization, Bone Mineral Density (BMD (mg/cm3)) was determined based on the linear correlation between CT attenuation coefficient and bone mineral density using a calibrated phantom.

### 2.2 Microscopic histologic evaluation

The condylar process of each hemi-mandible was separated at a height of about 5 mm. The right condyles were decalcified and the left condyles were used for undecalcified sections. Sectioning of the condyles was performed at mid sagittal plane.To this aim, three reference points using blue tissue marker dye (Shandon tissue marking dye Thermo scientific MI, USA) were marked directly on the condyle. These points were a. the anterior edge between the cartilage and bone, b. posterior edge point between the cartilage and bone, and c. midpoint of “a” and “b” on the upper most articular surface in the frontal dimension. Having these points on sections confirmed that they were at mid sagittal plane.

### 2.3 Alcian blue-pas staining

The right condyles were decalcified using Cal-ex II fixative-decalcifier (formaldehyde 1.03 M/L, formic acid 2.56 M/L) (CS511-4D, Fisher Scientific, Fair lawn, NJ, USA) for 3 weeks. After decalcification, the samples were embedded in paraffin and then serial sections were cut using a rotary microtome (Leica RM 2155; Wetzlar, Germany). Subsequent to dewaxing and hydrating in distilled water, the slides were placed in 3% acetic acid for 5 minutes and then in filtered Alcian Blue PH2.5 for 15 minutes. After rinsing in distilled water, the slides were placed in 0.5% periodic acid for 10 minutes and then washed well under tap then distilled water. Slides were placed in Schiff's reagent for 10 minutes, rinsed in distilled water for 5 minutes, stained in Harris Hematoxylin for 2 minutes and then washed well in tap water. Slides were dipped in 1% acid alcohol 3 times, and washed under tap water. The same procedure was performed in 1% Lithium Carbonate. Finally, the slides were dehydrated using 95% ethanol and 100% ethanol, mounted in Xylene and cover slipped using permount; a glass cover slip were added to the slides.

From each specimen, three slides were determined for evaluation. Photomicrographs were taken using a Leica fluorescent digital microscope with a CCD Digital camera (Leica, Wetzlar, Germany) and the image processing analyses were done using RS Image software (Version 1.73, Photometrics, Roper Scientific, Tucson, AZ, USA). The middle third of the posterior and middle regions of the condyle were evaluated (Fig. 2). The thickness of the fibrous, proliferative, and total layers as well as the cell population in proliferative and chondroblastic layers were measured. To quantify the bone remodeling activity [3,6] and the amount of active bone formation [7,8], bone volume fraction and percentage of newly formed bone and calcifying cartilage area / bone area were calculated respectively.

### 2.4 In vivo flourochrome labeling

The left specimens, following fixation in formaldehyde at RT, were rinsed in distilled water and transferred to 70% 2-propanol for 24 hrs. Samples were placed in an automated tissue processor for dehydration and clearing. Dehydration was performed through 2-propanol gradient of 80%, 95%, 95% and 100% with 4 hours for each step at RT. Clearing (defatting) was done by replacing 100% 2-propanol with two exchanges of methyl salicylate for 6 hrs. Infiltration and embedding the samples in Methyl Metacrylate was performed as follows: After removing the samples from processor they were placed in to a Methyl Methacrylate monomer for 24 hrs. Then, the monomer was removed and replaced with a Methyl Methacrylate mixture containing plasticizer and catalyst for the same duration. This was removed and replaced with a Methyl Methacrylate mixture containing plasticizer and an additional catalyst for 24 hrs. Finally, the mixture was removed and samples were embedded in glass vials with a Methyl Methacrylate embedding mixture containing plasticizer and catalyst till their polymerization was completed. In each procedure vacuum pressure was applied for 1hour in 20‐minute intervals. Polymerized blocks were broken out of the embedding vials, shaped and 6‐micron thick serial sections were cut at mid sagittal plane with a D‐profile tungsten carbide knife using a heavy‐duty rotary microtome. To assure that all the sections are at mid sagittal plane and similar for all the samples they were measured in all directions and the thickness was noted prior to embedding. While sectioning, trimming was performed until the mid-point was determined based on the thickness of each particular sample. Cut sections were adhered to pre‐cleaned, silane‐gelatin coated glass slides. Mounted sections were allowed to dry for 24 hours. Sections were deplasticized and cover slipped for fluorophore analysis.

From each of the left condyles, 3 slides were evaluated. The calcein green labels produced by in vivo flourochrome injection at 7 and 28 days of the experiment, were visualized under epifluorescence illumination and photomicrographs were taken using an Olympus Fluo View 1000 Inverted IX81 microscope. The image processing analyses were done using RS Image software (Version 1.73, Photometrics, Roper Scientific, Tucson, AZ, USA). Excitation and emission wavelength filter settings for visualizing calcein labels were respectively 436-495 nm and 517-540 nm. To estimate the amount of endochondral bone growth, the distance between the calcein labels which demarcates the mineralization front at time of administration [9] was measured in the middle region (Fig. 3).

### 2.5 Goldner's trichrome staining for osteoid

Two slides prepared from each undecalcified specimen, were deplasticized using Xylene and plastic film was removed. Then they were placed in warm (40-60°C), Cool (at RT, with agitation) and warm Xylene for 40, 20, and 20 minutes, respectively. In each step, Xylene was discarded after use. Slides were rehydrated through graded series of ethanol (EtOH): 100 % EtOH for 2–5 min, 100 % EtOH for 2–5 min, 95 % EtOH for 2–5 min, 70–80 % EtOH for 2–5 min, and distilled water (DI H2O) for 2–5 min. staining in a working solution of Weigert's iron hematoxylin for 15 min was also performed. Then slides were dipped in DI H2O, washed in gently running tap (basic pH) water for 15 min and again dipped in DI H2O.Slides were stained in a ponceau–acid fuchsin for 15 min. Following two times rinsing and shaking in 1 % acetic acid, they were quickly dipped in DI H2O to remove acid. The same procedure was performed after staining the slides in phosphomolybdic acid–Orange G for 8 min, and also, staining in Light Green SF Yellowish for 15 min. Then, slides were dehydrated through following graded ethanol immersions: 70–80 % EtOH for 2–5 min, 95 % EtOH for 2–5 min, 100 % EtOH for 2–5 min, and 100 % EtOH for 2–5 min, cleared in xylene for 2–5 min, and a cover slip was added.

Photomicrographs were taken using a Leica fluorescent digital microscope with a CCD Digital camera (Leica, Wetzlar, Germany) and the image processing analyses were done using RS Image software (Version 1.73, Photometrics, Roper Scientific, Tucson, AZ, USA). Evaluation was performed in the middle and posterior regions and the thickness of the osteoid was calculated (Fig. 4).

For all of the histomorphometric parameters, the average of measurements obtained from all the evaluated slides out of each sample was used as one registration for subsequent statistical analysis.

## Ethics Statement

This experiment was approved by the Animal Care and Use Committee for Health Sciences, University of Alberta, Canada (AUP: 000000381-REN1).

## CRediT Author Statements

**Yasamin Hadaegh:** Methodology, Visualization, Investigation, Data curation, Writing – original draft preparation; **Tarek El-Bialy:** Supervision Conceptualization, Validation, Reviewing and Editing.

## Declaration of Competing Interest

The authors declare that they have no known competing financial interests or personal relationships that could have appeared to influence the work reported in this paper.

## Data Availability

Mandibular Condyle Tissue Reaction to Low Intensity Pulsed Ultrasound in Young Adult Rats: Micro Computed Tomographic and Histomorphometric Datasets (Original data) (Mendeley Data).
